# A Survey of Knowledge, Approaches, and Practices Surrounding Parasitic Infections and Antiparasitic Drug Usage by Veterinarians in Türkiye

**DOI:** 10.3390/ani13172693

**Published:** 2023-08-23

**Authors:** Mahmut Sinan Erez, İlkay Doğan, Esma Kozan, Ahmet Göksu

**Affiliations:** 1 Department of Parasitology, Faculty of Veterinary Medicine, Afyon Kocatepe University, 03200 Afyonkarahisar, Türkiye; mserez@aku.edu.tr (M.S.E.);; 2 Department of Biostatistics, Faculty of Medicine, Gaziantep University, 27310 Gaziantep, Türkiye; 3 Eville & Jones, Leeds LS15 8ZB, UK

**Keywords:** antiparasitic drug usage, drug resistance, parasitic infections, survey, veterinarian, Türkiye

## Abstract

**Simple Summary:**

Parasitic infections are a significant problem in veterinary medicine, and their management requires an in-depth understanding of the parasite life cycle, host-parasite interactions, and the appropriate use of antiparasitic drugs. In this study, we aimed to investigate the knowledge, approaches, and practices of veterinarians in Türkiye regarding parasitic infections and antiparasitic drugs usage. Our study surveyed a diverse group of veterinarians working in various fields of veterinary medicine, including small animal, large animal, and mixed animal practices. The results of our study revealed that while veterinarians in Türkiye generally have a good understanding of parasitic infections and antiparasitic drugs, there are still gaps in their practice, particularly regarding testing anthelmintic resistance, using parasitic diagnostic methods, and attending conferences, symposia, and workshops. Overall, our study provides valuable insights into the current state of parasitic infections and anthelmintic resistance management in veterinary medicine in Türkiye. We believe that the findings of our study will help inform the development of more effective strategies for the prevention and treatment of parasitic infections in animals and contribute to the ongoing efforts to improve animal health and welfare in the region. Our results are also expected to raise awareness about anthelminthic resistance and parasite infections among veterinarians in Türkiye.

**Abstract:**

Despite a global background of increasing anthelmintic resistance in parasites, little is known about the current parasite control strategies adopted within the livestock industry in Türkiye. The aim of this survey is to identify the parasitic diseases encountered by veterinarians, the methods and drugs used for the diagnosis and treatment of these diseases, parasite control practices, and other related factors. This survey was conducted online between October 2018 and March 2019 with the participation of 607 veterinarians working in different areas from seven different geographical regions of Türkiye. A total of 29 questions were posed to the veterinarians in the online survey. As a result of this survey, it was determined that veterinarians should utilize laboratory methods more frequently for the detection and diagnosis of parasitic diseases and anthelmintic resistance. It was concluded that to effectively implement diagnosis, prevention, and control measures for parasitic diseases, field veterinarians need to establish closer relationships within academia and increase their participation in national and international conferences, symposia, and workshops where knowledge sharing and exchange take place. In conclusion, antiparasitic drug resistance has become increasingly important recently, and therefore measures taken to prevent the development of resistance should be increased.

## 1. Introduction

Parasitic diseases have been responsible for economic losses since humans began domesticating animals.

While sick animals are being raised at high costs, the decrease in production leads to both reduced profits and economic losses due to low productivity and deaths [1,2]. Today, despite all of the developments in the diagnosis, treatment, and prophylaxis of diseases, some parasites have high morbidity and mortality rates, especially in animals, and they can still cause environmental pollution and economic losses, and even pose significant risks to public health [3].

Ruminants and equids on pasture are often at risk of being infected with various parasites. Parasite control is essential to protect animal health and increase productivity [4]. The primary method of combating parasites to prevent pasture contamination is the use of antiparasitic drugs [5]. It was reported that factors such as timing errors in the use of antiparasitic drugs and incorrect drug selection may not only make the drugs ineffective in the control of parasites but also lead to the development of antiparasitic drug resistance [6,7,8]. Drugs used for the control of internal and external parasites in domestic animals are extremely important for animal and public health. The regular and periodic use of these drugs in accordance with the manufacturer’s recommendations prevents the development of resistance and thus ensures continuous protection [3].

For the effective prevention and control of parasitic diseases, it is necessary to have a good understanding of the characteristics, epidemiology, and life cycles of parasites, their modes of transmission, accurate diagnosis, appropriate treatment, and identification of sources to break the life cycle [9,10]. Obtaining information about knowledge (what is known), attitudes (what is believed), and practices (what is performed) is essential for identifying knowledge gaps, addressing needs, and understanding the factors and barriers that influence behaviors. Through the use of questionnaire surveys focused on the management practices of ruminant gastrointestinal nematodes, valuable insights have been gained into the factors that influence veterinarians’ behaviors in different specific regions. This understanding is vital in order to develop effective communication strategies and promote adherence to specialist advice. However, it is important to recognize that the factors driving veterinarians’ decisions regarding sustainable helminth management may differ depending on the particular society they are a part of [11,12,13,14]. The aim of this survey is to identify the parasitic diseases encountered by veterinarians, the methods and drugs used for the diagnosis and treatment of these diseases, parasite control practices, and other related factors through a survey.

## 2. Materials and Methods

### 2.1. Scope of This Study

**Figure 1:**
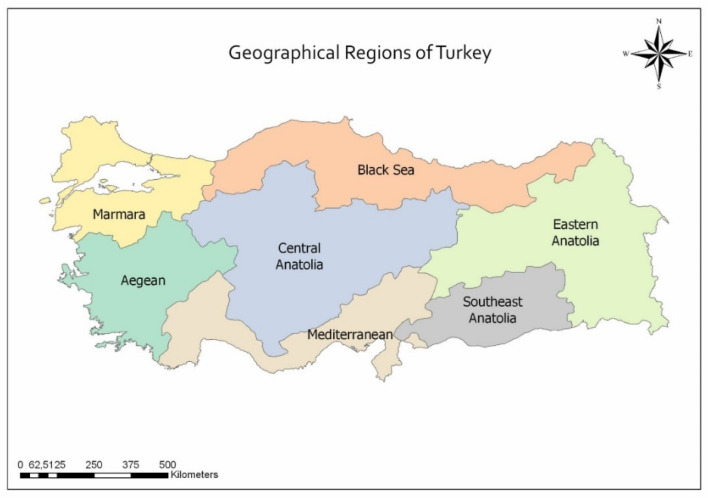
Geographical Regions of Türkiye (https://www.mappr.co/counties/turkey-regions/).

This study was conducted between October 2018 and March 2019 with 607 veterinarians from seven different geographical regions of Türkiye (Figure 1). These individuals were working in different fields such as academia, pet clinics, ruminant clinics, horse clinics, the government sector, and the industry sector.

### 2.2. Survey Design

A structured questionnaire was designed to collect data during an online survey. The questionnaire included 29 closed-ended questions with four open-ended options (Supplementary Materials). Study participants were recruited by posting an online invitation to participate by filling out an online questionnaire. The questionnaire was originally developed in English and was then translated into Turkish by one of the authors and pretested by sending to eight veterinarians based in different regions of Türkiye to evaluate its content and wording. The email addresses of veterinarians were obtained through regional chambers. A total of 2000 veterinarians were sent the survey and 607 responses were received. The questions were derived from a combination of common themes highlighted during discussions about parasitology with veterinarians in Türkiye. Additionally, the research group drew upon their own experience in parasite management and reviewed comparable literature on questionnaire-based studies related to parasitic infections and antiparasitic drug usage. The primary focus while developing the questions was to consider the areas of greatest importance to veterinarians across different working areas in terms of parasite control, antiparasitic drugs, anthelmintic resistance, and the diagnosis of parasitic infections.

The geographical regions in our country exhibit variations in terms of climate, natural features, human factors, economic conditions, and parasitic diseases. An equal number of veterinarians from each geographical area were recruited to participate in the survey. Additionally, veterinarians working in different fields, including large animal, companion animal, equine, government, and industry, were allowed to participate in the survey to assess their perspectives on parasitic diseases. The veterinarians who were included in this study were asked a total of 29 questions through an online survey regarding their age, gender, area and region of work, years of professional experience, parasitic diseases, and the parasite fauna of the region where they were working, their level of knowledge about control and prevention against parasitic diseases, use of parasitic diagnostic methods or level of utilization of faculties, institutes, or laboratories in the region for the diagnosis of parasitic diseases, participation in educational activities related to parasitic diseases, encountered parasitic diseases, decision-making processes for selecting and using drugs against these diseases, follow-up of treatment, and thoughts and experiences regarding antiparasitic drug resistance. The survey questions were modified from different survey studies [5,14,15,16,17].

### 2.3. Statistical Analysis

Descriptive statistics were used to analyze and outputs were presented in frequencies, proportions, and percentages for the analysis of questions related to participants’ demographic characteristics, the parasitic fauna of their working region, their knowledge levels about the prevention of parasitic diseases, their utilization of diagnostic methods for parasitic diseases or the level of benefit from faculties, institutes, or laboratories in the region for the diagnosis of parasitic diseases, their participation in educational activities related to parasitic diseases, the parasitic diseases they encountered, their decision-making processes regarding drug selection and drug usage for the treatment of parasitic diseases, and their opinions and experiences related to antiparasitic drug resistance. The relations between categorical variables were tested with Chi-square analysis (Likelihood ratio test). The analyses were conducted using SPSS 22.0 software and a significance level of *p* < 0.05 was chosen.

## 3. Results

The distributions of the participants based on their age, gender, years of professional practice, area of work, and region are given in Table 1.

It was determined that there was a statistically significant relationship (*p* < 0.05) between the working area of the participants and the region, gender, the frequency of participation in congresses, symposia, and educational seminars related to parasitic diseases, frequencies of using parasitic diagnostic methods, frequencies of utilizing the faculties, institutes, or laboratories in their region for parasitic diagnosis, and frequencies of monitoring newly emerging antiparasitic drugs (Table 2).

There was a statistically significant relationship (*p* < 0.05) between the age of the participants and their frequencies of following newly emerging antiparasitic drugs (Table 3).

Three hundred and seventeen (52.22%) participants reported that they had never attended any congresses, symposia, or educational seminars related to parasitic diseases, while seven (1.15%) reported that they attended these always, twenty-three (3.79%) said they attended frequently, ninety-five (15.65%) said they attended occasionally, and one hundred and sixty-five (27.18%) said they attended rarely. Parasitic diagnostic methods were never used by 242 (39.87%) of the participants, while these methods were always used by 25 (4.12%). In terms of diagnosis, the faculties, institutes, or laboratories in the region were always utilized by 14 participants, while they were frequently utilized by 54 (8.90%), occasionally utilized by 118 (19.44%), rarely utilized by 171 (28.17%), and never utilized by 250 (41.19%). Parasitic diseases that they could not diagnose were reported by 388 (63.92%) of the participants, while 219 (36.08%) of the participants stated that they had not encountered any parasitic disease that they could not diagnose. Antiparasitic drugs were reported to be frequently used on large animals by 42.34% (257) of the participants, on cats and dogs by 32.95% (200), on small ruminants by 21.09% (128), on poultry by 1.98% (12), on horses by 0.82% (5), and on other animals by 0.82% (5).

Gastrointestinal nematode infections, ectoparasite infestations, tapeworm infections, coccidiosis, cryptosporidiosis, liver flukes, ascarid infections, theileriosis, babesiosis, lungworm infections, heartworm, and rumen flukes were reported as the most commonly encountered parasitic diseases by the participants. Trichomoniasis, giardiasis, and varroosis were frequently encountered by some participants in addition to these diseases. The distributions of the factors taken into account by the participants in the selection of antiparasitic drugs are given in Table 4.

Antiparasitic drugs for prevention purposes were used routinely twice a year by 240 (39.54%) of the participants, oral medication applications were preferred by 297 (48.93%), and injectable medication applications were preferred by 260 (42.83%), based on their responses to the survey. It was reported that the effectiveness of an antiparasitic after usage was always monitored by 176 (29.00%) participants, frequently monitored by 255 (42.01%), and never monitored by 8 (1.32%). Newly released antiparasitic drugs were always followed by 118 (19.44%) participants, while they were never followed by 27 (4.45%). It was found that while the ineffectiveness of the drugs that were used was not considered by 40 (6.59%) participants, an opposing view was expressed by 9 participants, and 321 participants reported the occasional ineffectiveness of these drugs. When asked about their thoughts on the development of resistance to parasitic drugs, 81 (13.34%) always, 201 (33.11%) frequently, 213 (35.09%) occasionally, 82 (13.51%) rarely, and 30 (4.94%) never thought that resistance develops. The percentage distributions of measures applied by the participants to prevent the development of antiparasitic drug resistance are presented in Table 5.

Antiparasitic drugs can be obtained in Türkiye either with a prescription or without a prescription by those in need. Considering the development of resistance against antiparasitic drugs, it was stated by 358 (58.98%) of the participants that antiparasitic drugs should be sold with a prescription, while 111 (18.29%) reported that no prescription is necessary.

When queried as to whether they received feedback from animal owners to assess the efficacy of drugs at the desired level, 272 (44.81%) participants responded that they did so frequently, while 18 (2.97%) stated that they never asked for feedback.

The distributions of parasitic diseases for which the participants frequently administered antiparasitic treatments are given in Table 6.

Treatments for Ehrlichia, Giardia, Neospora, and *Oestrus ovis* were reported to be applied by the participants from time to time, in addition to treatments for the diseases listed in Table 6. It was determined that the most frequently used antiparasitic drugs by the participants were albendazole, oxfendazole, levamisole, oxyclozanide, praziquantel, ivermectin, doramectin, fipronil, and halofuginone.

## 4. Discussion

Veterinary education has been going on in Türkiye since 1842, and there are currently 33 veterinary faculties, 31 of which are actively providing education. Graduates of these faculties are equipped with the knowledge and skills to diagnose, treat, and control animal diseases, as well as to specialize in areas such as animal breeding, zoonotic diseases, and food safety. However, the educational curricula of these faculties need to be updated in response to various factors such as emerging technologies, the needs of the livestock sector, the emergence of new diseases, or the development of drug resistance. This study was conducted to assess the status of parasitic diseases, approaches to these diseases, and drug resistance among veterinarians working in different sectors.

Considering the ages of the veterinarians who participated in the survey, 55.19% of them were young veterinarians under the age of 30. The higher participation rate of young individuals in the interactive survey is believed to have stemmed from their greater utilization of technology compared to individuals over the age of 50. The higher interest in the survey by newly practicing veterinarians is believed to have occurred due to their effort to follow innovations, improve themselves, and stay up to date in their profession.

The livestock sector has become an important industry and an integral part of the economy in all parts of the world, especially in developed countries. While there were 10.7 million cattle in Türkiye in 2000, this figure reached 17.9 million in 2020. In 2010, there were 84.7 thousand buffalo, which increased 2.3 times and reached 192.8 thousand in 2020. In small ruminant livestock, the number of sheep increased by 82% to 42.1 million, and the number of goats increased by 90% to approximately 12 million in the last 10 years. In the poultry industry, there has been a significant increase in the number of modern poultry farms and integrated facilities, leading to notable increases in egg and white meat production [18]. Therefore, there is a growing need for veterinarians, especially in the field of ruminant diseases in Türkiye. The fact that 53.71% of the participants specialize in ruminant practice suggested that their career preferences were aligned with the needs of the industry. In recent years, there has been an increase in demand for pet animals in Türkiye, and keeping a pet at home has become popular. Although the opinions of experts have had a positive impact on the decisions of individuals to become pet owners, it is not known how accurately pet owners are informed about their animals and whether they implement the right practices [19]. In parallel with this development in the pet sector, the need for pet medicine has also increased, and in support of this, veterinarians practicing pet medicine ranked second at 23.23% participation in the survey. It was determined that there was a statistically significant relationship (*p* < 0.05) between the working area of the participants and the region they were working in. Accordingly, pet and horse clinics were mostly found in the Marmara region, and academicians were mostly working in the Aegean region. Furthermore, it was determined that there was a statistically significant relationship between the work area and gender of the participants (*p* < 0.05). It was found that small animal clinics were generally preferred by women, while ruminant clinics were preferred by men.

Conferences, symposia, and workshops that discuss current and professional topics and provide a platform for sharing knowledge with experienced individuals can significantly contribute to the professional development of newly graduated veterinarians. However, it was determined that 52.22% of the participants in this survey never attended congresses, symposia, or seminars organized on parasitic diseases, while only 1.15% said they always attended these. It was seen that there was a statistically significant relationship (*p* < 0.05) between the frequency of participation in congresses, symposia, and educational seminars related to parasitic diseases among the participants and their area of work. Accordingly, it was observed that equine clinic, government, and industry employees mostly did not participate in congresses, symposia, or training seminars on parasitic diseases. It has been suggested that the participation rate of veterinarians in such events should increase as continuous education contributes to the maintenance of professional knowledge and skills, awareness of new scientific developments, and addressing knowledge and experience deficiencies.

Currently, the detection and diagnosis of parasitic diseases is performed using various laboratory methods, considering the geographical region where the patient is located, clinical symptoms, anamnesis, and transportation information. While 25 (4.12%) of the participants in this study reported always using parasitic diagnostic methods, 242 (39.87%) stated that they never used these methods. A statistically significant relationship was determined between the work areas of the participants and their frequencies of using parasitic diagnostic methods (*p* < 0.05), whereby parasitic diagnostic methods were almost never used in ruminant clinics and frequently used in pet clinics. The faculties, institutes, or laboratories in their region of practice were always utilized by 14 participants for parasitic diagnosis, while 54 (8.90%) frequently utilized them, 118 (19.44%) occasionally utilized them, 171 (28.17%) rarely utilized them, and 250 (41.19%) reported that they had never used these resources. A statistically significant relationship was found between the working areas of the participants and their frequencies of utilizing the faculties, institutes, or laboratories in their region for parasitic diagnosis (*p* < 0.05). Ruminant clinics almost never utilized the faculties, institutes, or laboratories in the region for parasitic diagnosis. It was reported that parasitic diseases that could not be diagnosed were encountered by 388 (63.92%) of the participants, while 219 (36.08%) participants said there was no parasitic disease that they could not diagnose. The accurate diagnosis of the etiology of parasites and the implementation of appropriate treatment methods are crucial in combating parasitic diseases. According to the survey results, the participants did not frequently use parasitic diagnostic methods, and they did not seek much assistance from experts in cases where they were unable to diagnose diseases. This situation may pose a barrier to effectively combating parasitic diseases. Accurate diagnosis and the appropriate administration of antiparasitic drugs are crucial for effectively managing parasitic diseases. Nevertheless, the survey findings reveal room for improvement in terms of diagnostic practices, indicating a potential barrier to comprehensively addressing parasitic diseases. According to the information provided by the participants in this survey, the administration of antiparasitic drugs to large livestock animals is the most common practice, followed by cats and dogs. It was reported that gastrointestinal nematodes, tapeworms, ectoparasites, and coccidiosis pathogens were the most commonly encountered parasites, while parasites such as rumen flukes and heartworm were encountered less frequently. In addition to the reported parasites, some participants reported Trichomonas, Giardia, and Varroa. The drugs frequently used by the participants were compatible in terms of their effect on the most common parasitic diseases.

Taking preventive measures before the emergence of diseases, within the scope of preventive veterinary medicine, is the most practical and economical way to protect animals from diseases. Preventive medicine is possible through antiparasitic measures of endo- and ecto-parasites before the disease appears in the area or in the animal [20,21,22]. In this context, 39.54% of the participants stated that they used antiparasitic drugs for preventive purposes at least twice a year. The irresponsible and irregular administration of anthelmintic drugs hinders effective control and leads to the development of resistance to drugs. Therefore, the possibility of resistance to drugs in parasites should not be overlooked. In general, antiparasitic resistance refers to the development of heritable insensitivity over time in the same population of parasites to an antiparasitic drug that can eliminate a large portion of the parasite population in the host when applied at the recommended dose [23,24,25]. Appropriate antiparasitic selection, avoiding the unnecessary use of drugs, testing for the development of resistance, using narrow-spectrum drugs, and taking into account factors such as climate and geographical conditions are highly effective in preventing the development of antiparasitic resistance [26,27,28]. Since the consecutive and frequent use of drugs from the same group can lead to the development of resistance, it is necessary to have protocols for monitoring newly emerging antiparasitic drugs and to use different groups of drugs. According to the results of this survey, a statistically significant relationship (*p* < 0.05) was detected between the specialization areas of the participants and their frequencies of monitoring newly emerging antiparasitic drugs. In this context, it was concluded that while ruminant veterinarians monitored newly emerging antiparasitic drugs, government and industry veterinarians did not follow the drug market. Additionally, there was a statistically significant relationship between the age of the participants and their frequencies of following newly emerging antiparasitic drugs (*p* < 0.05). Thus, it was determined that most of the young participants who are inexperienced in veterinary practice did not follow new antiparasitic drugs, and this situation was evaluated as a deficiency for young veterinarians.

It was observed that the participants were aware of antiparasitic drug resistance. Nonetheless, while 69.03% of the participants did not frequently use the same active ingredient to prevent resistance development, only 5.11% conducted the fecal egg count reduction test, which is a highly convenient and effective method for detecting resistance development. In various studies [23,29,30,31], it has been reported that resistance has developed against some drugs in Türkiye. This has led to the belief that measures taken by veterinarians to prevent resistance are inadequate. Veterinarians need to be supported in conducting a cost-effective and practical test called the fecal egg count reduction test for detecting resistance development.

This study sheds light on the state of veterinary practice in Türkiye, particularly with regard to parasitic diseases and their management and emphasizes the significance of knowledge sharing and continuous education in effectively addressing parasitic diseases.

## 5. Conclusions

In conclusion, this survey study is a reflection of the field regarding this topic for the first time in Türkiye, the preferences of veterinarians in their fields of work, the parasitic diseases found, the methods and drugs they used for the diagnosis and treatment of these diseases, parasite control practices, and other related factors. While participants are aware of drug resistance, there is a need for more proactive approaches, such as regular monitoring of emerging antiparasitic drugs and the adoption of resistance detection methods. Regarding diagnostics and treatment, this study highlights variations in the utilization of parasitic diagnostic methods among different sectors, with ruminant clinics displaying lower utilization compared to pet clinics. It is believed that field veterinarians need to have closer relationships within academia to effectively implement the diagnosis, prevention, and control measures of parasitic diseases, and their participation in national and international meetings such as conferences, symposia, and workshops for knowledge sharing should increase. Recently, antiparasitic drug resistance has become very important, so it is thought that measures to be taken to prevent the development of resistance should be increased. It is thought that this survey study may contribute to the evaluation and improvement of parasitology education in veterinary medicine in Türkiye.

## Figures and Tables

**Table 1:** Distributions of participants by age, gender, years of practice, area of practice, and region of practice.

		Number (n)	%
Age	21–30	335	55.2
	31–40	185	30.5
	41–50	68	11.2
	>50	19	3.1
Gender	♂	544	89.6
	♀	63	10.3
Years of Practice	0–4	286	47.1
	5–9	165	27.2
	>10	156	25.7
Area of Practice	Academy	25	4.1
	Pet Clinics	141	23.2
	Ruminant Clinics	326	53.7
	Horse Clinics	6	0.99
	Public Veterinarian	88	14.5
	Industry	21	3.46
Region of Practice	Marmara	115	18.9
	Central Anatolia	125	20.6
	Aegean	165	27.2
	Eastern Anatolia	53	8.7
	Southeastern Anatolia	46	7.6
	Mediterranean	56	9.2
	Black Sea	47	7.7

**Table 2:** A Comparative Analysis of Working Area and Selected Variables.

Variables		Working Area						*p*
		Academy	Pet Clinics	Ruminant Clinics	Horse Clinics	Public Veterinarian	Industry	
		N (%)	N (%)	N (%)	N (%)	N (%)	N (%)	
Gender	Female	6 (24)	36 (25.53)	9 (2.76)	2 (33.33)	5 (5.68)	5 (23.81)	0.001 *
	Male	19 (76)	105 (74.47)	317 (97.24)	4 (66.67)	83 (94.32)	16 (76.19)	
Region	Marmara	3 (12)	55 (39.01)	35 (10.74)	3 (50)	12 (13.64)	7 (33.33)	0.001 *
	Central A.	5 (20)	27 (19.15)	73 (22.39)	1 (16.67)	16 (18.18)	3 (14.29)	
	Aegean	10 (40)	39 (27.66)	86 (26.38)	1 (16.67)	25 (28.41)	4 (19.05)	
	Eastern A.	4 (16)	0 (0)	37 (11.35)	1 (16.67)	11 (12.5)	0 (0)	
	Southeastern A.	1 (4)	0 (0)	33 (10.12)	0 (0)	10 (11.36)	2 (9.52)	
	Mediterranean	1 (4)	15 (10.64)	30 (9.2)	0 (0)	8 (9.09)	2 (9.52)	
	Black Sea	1 (4)	5 (3.55)	32 (9.82)	0 (0)	6 (6.82)	3 (14.29)	
Frequency of utilizing parasitic diagnostic methods	Always	5 (20)	17 (12.06)	2 (0.61)	0 (0)	1 (1.14)	0 (0)	0.001 *
	Often	4 (16)	44 (31.21)	13 (3.99)	0 (0)	3 (3.41)	2 (9.52)	
	Occasionally	4 (16)	34 (24.11)	54 (16.56)	4 (66.67)	16 (18.18)	3 (14.29)	
	Rarely	5 (20)	30 (21.28)	93 (28.53)	2 (33.33)	26 (29.55)	3 (14.29)	
	Never	7 (28)	16 (11.35)	164 (50.31)	0 (0)	42 (47.73)	13 (61.9)	
Utilization frequency of local faculties, institutes, and laboratories for parasitic diagnosis	Always	4 (16)	4 (2.84)	4 (1.23)	0 (0)	2 (2.27)	0 (0)	0.001 *
	Often	8 (32)	20 (14.18)	12 (3.68)	2 (33.33)	11 (12.5)	1 (4.76)	
	Occasionally	1 (4)	39 (27.66)	53 (16.26)	1 (16.67)	22 (25)	2 (9.52)	
	Rarely	3 (12)	40 (28.37)	92 (28.22)	3 (50)	25 (28.41)	8 (38.1)	
	Never	9 (36)	38 (26.95)	165 (50.61)	0 (0)	28 (31.82)	10 (47.62)	
Frequency of participation in congresses, symposiums and training seminars on parasitic diseases	Always	2 (8)	4 (2.84)	1 (0.31)	0 (0)	0 (0)	0 (0)	0.001 *
	Often	4 (16)	12 (8.51)	3 (0.92)	1 (16.67)	1 (1.14)	2 (9.52)	
	Occasionally	2 (8)	30 (21.28)	43 (13.19)	2 (33.33)	16 (18.18)	2 (9.52)	
	Rarely	4 (16)	42 (29.79)	90 (27.61)	1 (16.67)	21 (23.86)	7 (33.33)	
	Never	13 (52)	53 (37.59)	189 (57.98)	2 (33.33)	50 (56.82)	10 (47.62)	
Following new antiparasitic drugs	Always	5 (20)	40 (28.37)	63 (19.33)	1 (16.67)	6 (6.82)	3 (14.29)	0.001 *
	Often	4 (16)	58 (41.13)	105 (32.21)	3 (50)	22 (25)	5 (23.81)	
	Occasionally	9 (36)	35 (24.82)	110 (33.74)	1 (16.67)	38 (43.18)	5 (23.81)	
	Rarely	5 (20)	7 (4.96)	38 (11.66)	1 (16.67)	12 (13.64)	4 (19.05)	
	Never	2 (8)	1 (0.71)	10 (3.07)	0 (0)	10 (11.36)	4 (19.05)	

* *p* < 0.05; Likelihood ratio test.

**Table 3:** Comparison of age and following new antiparasitic drugs.

		Following New Antiparasitic Drugs					
Variables		Always	Often	Occasionally	Rarely	Never	*p*
		N (%)	N (%)	N (%)	N (%)	N (%)	
Age	21–30	58 (49.15)	104 (52.79)	113 (57.07)	45 (67.16)	15 (55.56)	0.011 *
	31–40	35 (29.66)	60 (30.46)	66 (33.33)	16 (23.88)	8 (29.63)	
	41–50	14 (11.86)	27 (13.71)	18 (9.09)	5 (7.46)	4 (14.81)	
	>50	11 (9.32)	6 (3.05)	1 (0.51)	1 (1.49)	0 (0)	

* *p* < 0.05; Likelihood ratio test.

**Table 4:** Distributions of factors considered by veterinarians in antiparasitic drug selection.

	Always		Occasionally		Never	
	Number (n)	Percentage (%)	Number (n)	Percentage (%)	Number (n)	Percentage (%)
Targeted parasite	509	83.85	87	14.33	11	1.8
Number of animals treated	350	57.66	157	25.86	100	16.5
Ease of drug administration	380	62.60	175	28.84	52	8.5
Brand of the medicine	254	41.85	250	41.19	103	16.9
Diagnosis results	364	59.97	153	25.20	90	14.8
Period of drug excretion	170	28.01	230	37.89	207	34.1
Experiences/requests of the patient owner	113	18.61	292	48.11	202	33.3
Drug price	200	32.95	295	48.60	112	18.4
Your experience	483	79.57	110	18.13	14	2.3
Duration of drug action	384	63.26	175	28.83	48	7.9

**Table 5:** Percentage distributions of measures applied by the participants to prevent the development of drug resistance.

Measure to Prevent the Development of Resistance	Number (n)	Percentage (%)
Not using the same active ingredient frequently	419	69.0
Conducting a fecal egg count reduction test	31	5.1
Avoiding the unnecessary use of antiparasitic drugs	127	20.9
Considering the development of concomitant immunity	57	9.4
Applying treatment according to age	89	14.7

**Table 6:** Distributions of parasitic diseases for which the participants frequently applied antiparasitic treatment.

Parasitic Disease	Number (n)	Percentage (%)
Gastrointestinal nematode infection	321	52.9
Liver fluke infection	169	27.8
Rumen fluke infection	20	3.3
Ascarid infection	159	26.2
Tapeworm infection	267	44
Heartworm infection	20	3.3
Lungworm infection	101	16.6
Babesiosis	62	10.2
Theileriosis	69	11.4
Cryptosporidiosis	144	23.7
Coccidiosis	181	29.8
Ectoparasite infestation	319	52.5

## Data Availability

The data presented in this study are available on request from the corresponding author.

## Supplementary Materials

The following supporting information can be downloaded at: https://www.mdpi.com/article/10.3390/ani13172693/s1, Survey Questions.
