# Supplementary material for: A Survey of Knowledge, Approaches, and Practices Surrounding Parasitic Infections and Antiparasitic Drug Usage by Veterinarians in Türkiye
Source: Animals (Basel). 2023 Aug 23;13(17):2693. doi: 10.3390/ani13172693 (PMC10486779; doi:10.3390/ani13172693)
Supplement: Supplementary file 1 [file animals-13-02693-s001.zip › animals-2493986-SI.pdf]

Survey Questions

1. Your age:

(1) 21-30 years (2) 31-40 (3) 41-50 (4) Over 50

2. 2. Your gender:

(1) Female (2) Male

3. How many years have you been practicing this profession?

(1) 0-4 years (2) 5-9 years (3) 10 years and above

4. Your field of work :

(1) Academy (2) Pet Clinic (3) Cattle Clinic (4) Horse Clinic (5) Government (6) Industry (7) Other

5. The region where you work

(1) Marmara Region (2) Central Anatolia Region (3) Aegean Region (4) Eastern Anatolia Region

(5) South Eastern Anatolia Region (6) Mediterranean Region (7) Black Sea Region

6. Your level of knowledge about the parasite fauna in your working region:

(1) Very good (2) Good (3) Moderate (4) Insufficient (5) None

7. Your level of knowledge about parasitic infections:

(1) Very good (2) Good (3) Moderate (4) Insufficient (5) None

8. Your level of knowledge about prevention and control of parasitic infections:

(1) Very good (2) Good (3) Moderate (4) Insufficient (5) None

9. The frequency of using parasitic diagnostic methods (Flotation, Sedimentation, Baermann-Wetzel, blood tests, etc.):

(1) Always (2) Often (3) Occasionally (4) Rarely (5) Never

10. The frequency of utilizing faculties, institutes, or laboratories in your region for parasitic diagnosis:

(1) Always (2) Often (3) Occasionally (4) Rarely (5) Never

11. The frequency of attending congresses, symposiums, and training seminars related to parasitic diseases:

(1) Always (2) Often (3) Occasionally (4) Rarely (5) Never

12. The most common parasitic infection(s) you encounter:

( ) Intestinal roundworms

( ) Liver flukes

( ) Rumen fluke

( ) Ascaris infections

( ) Tapeworm infections

( ) Heartworm

( ) Lung flukes

( ) Babesiosis

( ) Theileriosis

( ) Cryptosporidiosis

( ) Coccidiosis

( ) Infestations with ectoparasites

( ) Other (Please specify: _________________)

13. Did you encounter a parasitic infection that you couldn't diagnose?

(1) Yes (2) No

14. Species of animals for which you frequently use antiparasitic medication:

(1) Cattle

(2) Sheep and goats

(3) Horses

(4) Cats and dogs

(5) Poultry

(6) Other

15. The way you make decisions regarding the choice of antiparasitic drugs:

( ) Based on recommendations from other veterinarians

( ) Based on academic knowledge

( ) Based on current articles and books

( ) Based on advertisements and promotions

( ) Based on feedback from animal owners regarding previously used drugs

16. The factors you consider when choosing antiparasitic drugs:

( ) Always/Often ( ) Occasionally ( ) Rarely/Never

Targeted parasite

Number of animals to be treated

Ease of administration of the drug

Brand of the drug

Diagnostic results

Drug elimination period

Owner's experiences/wishes

Drug price

Your experience

Duration of drug efficacy

17. Do you have any frequently used antiparasitic drugs?

( ) Yes

( ) No

18. How do you prefer to administer the antiparasitic medication?

(1) Oral

(2) Injectable

(3) Topical (pour-on)

(4) Other (please specify)...

19. How often do you use antiparasitic medication for routine prevention?

(1) Once a year

(2) Twice a year

(3) Three times a year

(4) Four times a year

(5) Five times or more a year

20. Commonly treated parasitic infections with antiparasitic medication: (0/1)

( ) Gastrointestinal roundworms

( ) Liver flukes

( ) Rumen flukes

( ) Ascarid infections

( ) Tapeworm infections

( ) Heartworm

( ) Lungworms

( ) Babesiosis

( ) Theileriosis

( ) Cryptosporidiosis

( ) Coccidiosis

( ) Infestations with external parasites

( ) Other (please specify)..

21. Do you monitor the effectiveness of the antiparasitic medication after its use?

(1) Always

(2) Frequently

(3) Occasionally

(4) Rarely

(5) Never

22. Do you keep up with newly developed antiparasitic medications?

(1) Always

(2) Frequently

(3) Occasionally

(4) Rarely

(5) Never

23. Do you think the antiparasitic medication you use is ineffective?

(1) Always

(2) Frequently

(3) Occasionally

(4) Rarely

(5) Never

24. Do you believe that parasites have developed resistance to medications?

(1) Always

(2) Frequently

(3) Occasionally

(4) Rarely

(5) Never

25. What precautions do you take if you believe there is development of drug resistance? (0/1)

( ) I avoid using the same active ingredient frequently.

( ) I conduct fecal egg count reduction tests.

( ) I only use antiparasitic medication when necessary.

( ) I consider the development of concomitant immunity.

( ) I apply treatment approaches based on age (such as minimizing treatment in adult animals).

26. Do you believe you have sufficient knowledge and experience regarding the prevention and control of parasitic infections?

(1) Always

(2) Frequently

(3) Occasionally

(4) Rarely

(5) Never

27. Do you use two or more antiparasitic medications simultaneously?

(1) Always

(2) Frequently

(3) Occasionally

(4) Rarely

(5) Never

If yes, which active ingredients do you primarily use in combination?

....

28. How do you think antiparasitic medications should be sold?

(1) Over-the-counter

(2) Prescription-only

(3) It doesn't matter

29. Do you gather information from animal owners regarding the effectiveness or ineffectiveness of the medication?

(1) Always

(2) Frequently

(3) Occasionally

(4) Rarely

(5) Never
